# How Middle and High School Students Wear Their Face Masks in Classrooms and School Buildings

**DOI:** 10.3390/healthcare10091641

**Published:** 2022-08-28

**Authors:** Gerald Jarnig, Reinhold Kerbl, Mireille N. M. van Poppel

**Affiliations:** 1 Institute of Human Movement Science, Sport and Health, University of Graz, 8010 Graz, Austria; 2 Department of Pediatrics and Adolescent Medicine, LKH Hochsteiermark, 8700 Leoben, Austria

**Keywords:** COVID-19, face mask, wearing, school, classes, students, middle school, high school, school children, mitigation measures

## Abstract

In addition to other mitigation measures, face masks have been used in schools worldwide as a precondition for allowing school attendance during the COVID-19 pandemic. The quality and habits of mask wearing have, however, not been evaluated thus far, leaving uncertainty about the efficacy of this measure. It was the aim of this study to assess the accuracy of face mask wearing by children and adolescents in different school situations. In May and June 2022, students of two selected Austrian schools were asked to provide information about the different variations in wearing a face mask in different situations at school (in classrooms with or without the presence of a teacher, and in school buildings outside classrooms without the presence of a teacher). Strongly divergent results were identified for the amount of time in which face masks were worn correctly in the three different situations (*p* < 0.001, eta = 0.29). In the presence of a teacher, masks were worn correctly 63.7% of the time, while this percentage decreased to 31.9% when no teacher was present (*p* < 0.001). These results suggest the limited efficacy of mandatory face masks in schools. Should this measure become necessary again in the future due to the pandemic situation and highly pathogenic variants, special efforts are necessary in order to improve the quality of face mask wearing by school children.

## 1. Introduction

Beginning in late 2019, the rapid spread and life-threatening consequences of the COVID-19 pandemic [1] led to the use and prescription of various pharmaceutical and nonpharmaceutical measures worldwide in order to contain the infectious dynamics of the severe acute respiratory syndrome, coronavirus 2 (SARS-CoV-2) [2,3]. For children, adolescents, and adults worldwide, wearing a face mask was one of the nonpharmaceutical COVID-19 mitigation measures [4,5,6,7].

The practicality and usefulness of wearing medical or general face masks used to contain the spread of different virus variants have repeatedly been proven [8,9,10,11,12].

For groups in small spaces (i.e., children in school classrooms), wearing a face mask has been described as an effective way of limiting the transmission of SARS-CoV-2 [13,14], but it is also essential that classrooms are adequately ventilated [15]. However, there are also studies reporting problems that limit and complicate the correct wearing and use of face masks among children and adolescents, such as face shape, sense of responsibility, or hygiene [16,17,18,19].

Due to the ongoing COVID-19 pandemic and the associated high infection rates caused by the SARS-CoV-2 variants, Delta and Omicron, the wearing of face masks was temporarily mandatory in Austrian schools in the school year 2021/2022. During the period from November 2021 to the end of February 2022, students in secondary school were mandated to wear face masks, in addition to other mitigation measures (distance learning, SARS-CoV-2 testing, etc.) [20,21,22]. Beginning in March 2022, these mitigation measures were relaxed in stages due to reduced infection rates, and from then on, wearing a face mask was only mandated in school outside the classroom [23]. 

The aim of this study was to assess whether face masks were worn correctly by students in different situations at school, in order to obtain an impression of the usefulness and efficacy of mandatory face mask use in schools.

## 2. Materials and Methods

### 2.1. Design

In this retrospective cohort study, we compared variations in mask wearing habits in different situations at schools. The study was registered with the German Clinical Trials Registry (ID DRKS00029061) and approved by the Research Ethics Committee of the University of Graz, Styria, Austria (GZ. 39/70/63 ex 2021/22).

### 2.2. Selection of Schools and Participations

A semi-structured questionnaire was used at two school campuses in Austria (Salzburg and Carinthia) to investigate different variations in face mask wearing as perceived by students in different situations at schools.

**Figure 1:**
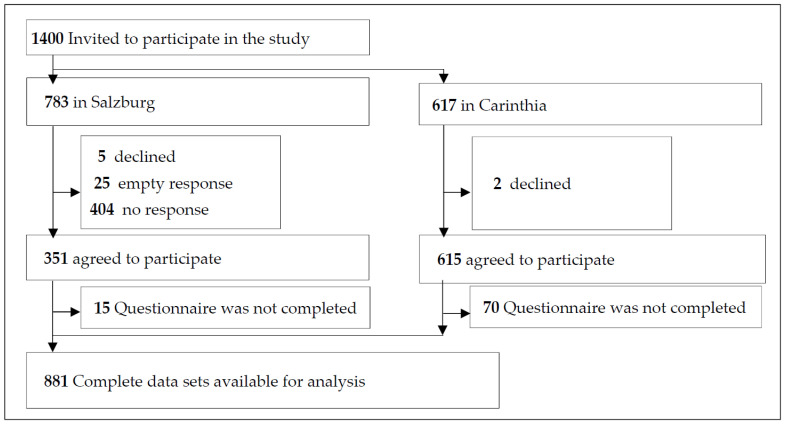
Study flow diagram.

A total of 1400 students (783 from Salzburg and 617 from Carinthia) were invited to participate in the study. They were asked to provide information about their perception of different variations in face mask wearing in different school situations. A total of 881 students (62.9% (Salzburg: 366 = 42.9%; Carinthia: 545 = 88.3%)) agreed to participate in the study and completed the questionnaire. For children younger than 14 years old, their legal guardians gave their written consent for their participation (Table S1, Figure 1)

At the two school campuses, individual students attended two different school branches, including a general school branch (GB) and a school branch with a sports focus (SF). Students in the SF branch were allowed to participate in sports and other physical activities without wearing a face mask and without other restrictions, according to the guidelines and safety measures for competitive sports in Austria [20], while students attending the GB branch were allowed to participate in physical activities and sports only under strict restrictions, in compliance with existing COVID-19 measures [21,22,23], which included wearing a face mask.

All of the students were either attending a middle school (middle school (M.S.): age 10–16 years, mean: 12.8 (95% CI = 12.7–13.0 years)) or a 4-year high school (high school (H.S.): age 14–20 years, mean: 16.7 (95% CI = 16.6–16.8 years)) at both of the school campuses (Table S1).

### 2.3. Procedures

In May and June 2022, the study participants were asked to provide information about the percentage of time that masks were worn with different variations in different situations at school.

Three different school situations were investigated, including two referring to the time spent in classrooms, and one referring to the time spent in school buildings outside the classroom. The time spent in the classroom was separated into the time spent in the classroom without a teacher present (I.c. -T) and the time spent in the classroom with a teacher present (I.c. +T). The time spent in school buildings outside the classroom was defined as the time spent outside the classroom without a teacher present (S.b. -T).

The students were asked to estimate the percentage of time that they had been wearing masks in school for each individual face mask wearing variant. The students had the opportunity to assign different percentage values for four different face mask wearing variants. For this purpose, they could choose one of the five following response options: either 0%, 25%, 50%, 75%, or 100% for each variant (see Methods S1). The students were also informed that they were allowed to offer self-estimated percentages in the questionnaire. Additionally, students were informed that the sum of the percentages of the four variants should equal 100% and which method would be used to convert the data should the sum exceed 100%.

For the self-evaluation, one variant denoting correct face mask wearing (variant 1 (V1) = mouth and nose covered) and three variants denoting incorrect face mask wearing (variant 2 (V2) = mouth uncovered, nose covered; variant 3 (V3) = mouth and nose uncovered; and variant 4 (V4) = mouth covered, nose uncovered) were defined (see Methods, S2). Students were asked to perform this self-evaluation for each of the three situations at school. These different situations at school were described in the questionnaire, and the different face mask wearing variants were illustrated with drawings (Methods S1). 

### 2.4. Outcomes

The primary outcome of this study was the identification of differences regarding the correct and incorrect wearing of face masks in classroom settings and school buildings in different situations in schools.

Secondary analyses were conducted for the subgroups by class membership and sex.

If the sum of the four estimated percentages resulted in a value of more or less than 100%, the results were adjusted downward or upward, keeping the ratios between the constant of the four variations. (For example, if the self-estimated percentages of a study participant: V1 = 50%, V2 = 75%, V3 = 50%, and V4 = 0%, giving 175% in total, the corrected results when the ratios remained the same were V1 = 28.6%, V2 = 42.9%, V3 = 28.6%, and V4 = 0.0%, giving 100% in total.)

### 2.5. Standardization

The continuous variables were reported as means (M) and standard deviations (SD), while the categorical variables were reported as absolute values (n) and percentages (%), for the descriptive statistics. No imputation of the data was performed.

The percentage of time spent correctly wearing masks (V1) was analyzed using a 3-way analysis of variance with repeated measures. Sex and school class membership were included in the models as between-participant effects, and different situations in the schools were included as within-participant effects.

For each situation in the schools, the percentages of time spent incorrectly wearing masks (V2, V3, and V4) were analyzed using a 3-way analysis of variance with repeated measures. Sex and school class membership were included in the models as between-participant effects, and different mask wearing variants (V2, V3, and V4) were included as within-participant effects.

In addition, the percentage of time spent incorrectly wearing masks was analyzed for each variant (V2, V3, and V4) in the different situations at school using a 3-fold analysis of variance with repeated measurements. Sex and school class were included in the models as between-participant effects, and different situations in the schools were included as within-participant effects.

In the case of non-sphericity, a Greenhouse–Geisser correction was performed. The α level correction for the post hoc tests was performed using the Bonferroni correction. For the analysis of variance, partial η^2^ (η_p_^2^) was used to determine the size of the effect (≥0.01, small; ≥0.06, medium; and ≥0.14, large); thus, small and large effects were considered relevant.

For the comparison of the results between two groups, a paired t-test was performed. All of the tests were two-tailed, and a *p*-value < 0.05 was considered statistically significant. 

All of the statistical calculations were performed using SPSS Version 27 (IBM Corp., released 2021, IBM SPSS Statistics for Windows, Armonk, NY, USA: IBM Corp).

## 3. Results

Among the 881 students (age: 15.1 ± 2.3 years, 43.2% female) included in this analysis, 330 students (37.5%) attended school classes with a sports focus (SF). The percentage of girls in the SF classes was significantly lower than that in the GB classes (SF: ♀ = 22.1%, GB: ♀ = 49.4%; *p* < 0.001). The students in the SF classes were slightly younger than those in the GB classes (SF: 14.7 years; GB: 15.3 years; *p* < 0.001) (Table S2). 

Depending on the situation in the schools, the percentage of correct face mask wearing varied. Students reported a significantly (*p* < 0.001) lower percentage of time wearing face masks correctly in classrooms without a teacher present (31.9%) compared to classrooms with a teacher present (63.7%). This difference was highly significant in all of the subgroups (*p* < 0.001) (Table 1 and Table S3).

For the time spent in school buildings outside the classroom and without a teacher present (S.b. -T), students reported higher rates of correct face mask use (57.9%, *p* < 0.001) compared to the time spent in classrooms without a teacher present. This difference was highly significant in all of the subgroups (*p* < 0.001) (Table 1 and Table S3).

When comparing the student-reported time of correct mask wearing in school buildings outside the classroom and without a teacher present with time spent in classrooms with a teacher present, highly significant (*p* < 0.001) differences were found. These differences were significant in all subgroups except the group of students in high school with a sports focus (in class with teacher = 52.7% versus outside classroom without teacher = 50.7%, *p* = 0.38) (Table 1 and Table S3). 

### 3.1. Correct Mask Wearing

**Figure 2:**
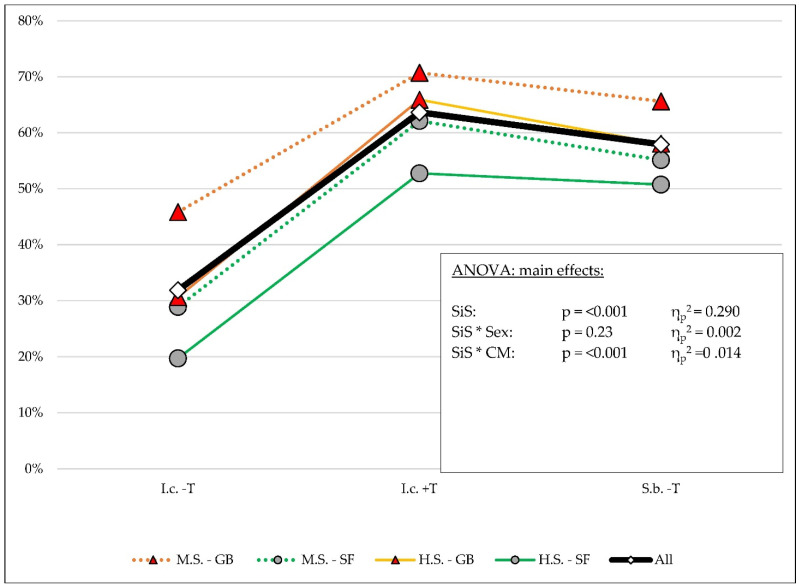
Percentage of self-estimated time of correct mask wearing with respect to the main effects in different situations in the schools.

**Figure 3:**
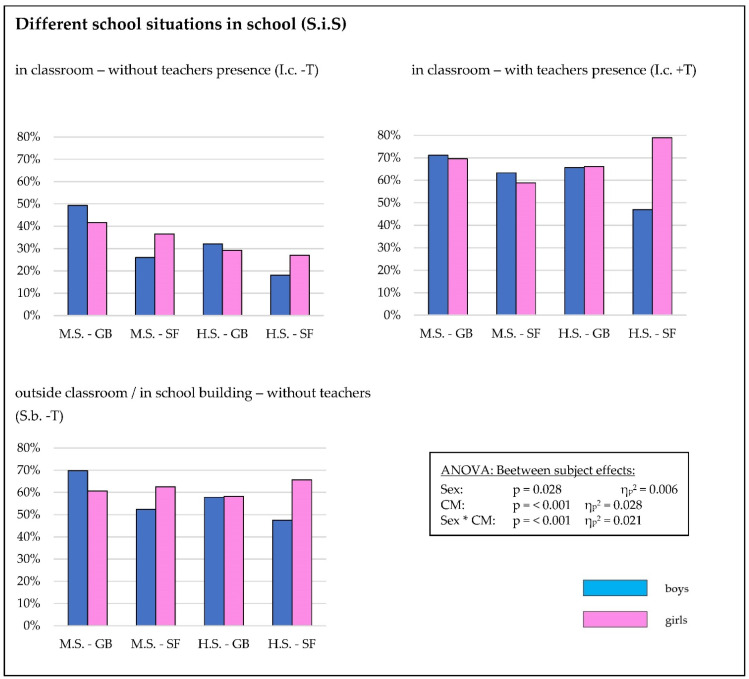
Detailed percentage of self-estimated correct mask wearing for the CM subgroups (class membership: M.S. GB, M.S. SF, H.S. GB, and H.S. SF) and sex (♂ = boys, ♀ = girls).

We observed a highly significant difference with a great effect when analyzing the correct wearing of face masks in different situations at school (main effect: I.c. -T: 31.9%; I.c. +T: 63.7%; and S.b. -T: 57.9%; *p* < 0.001, η_p_^2^ = 0.290). Interaction effects between the situations in the schools and class membership (*p* < 0.001, η_p_^2^ = 0.014) were found. No interaction effects were found with sex (*p* = 0.23, η_p_^2^ = 0.002) (Table 1 and Table 2, Figure 2 and Figure 3, and Tables S3–S5).

We found significant differences between several subgroups (*p* < 0.001, η_p_^2^ = 0.028). Students attending the middle school GB reported a significantly higher percentage of correct mask wearing compared to their counterparts of other class memberships (M.S. GB vs. M.S. SF: *p* = 0.003, M.S. GB vs. H.S. GB: *p* < 0.001, and M.S. GB vs. M.S. SF: *p* < 0.001). No differences were found when comparing the results of the students of the remaining class memberships (Table 1 and Table 2, Figure 3, and Tables S3, S4 and S6). 

We found a significant interaction between sex and class membership (Sex*CM: *p* < 0.001, η_p_^2^ = 0.021) (Table 1 and Table 2, Figure 3, and Tables S3 and S4).

Additional information about the class membership differences in the different situations in schools is reported in the Supplementary Materials (Table S7).

### 3.2. Incorrect Mask Wearing

**Figure 4:**
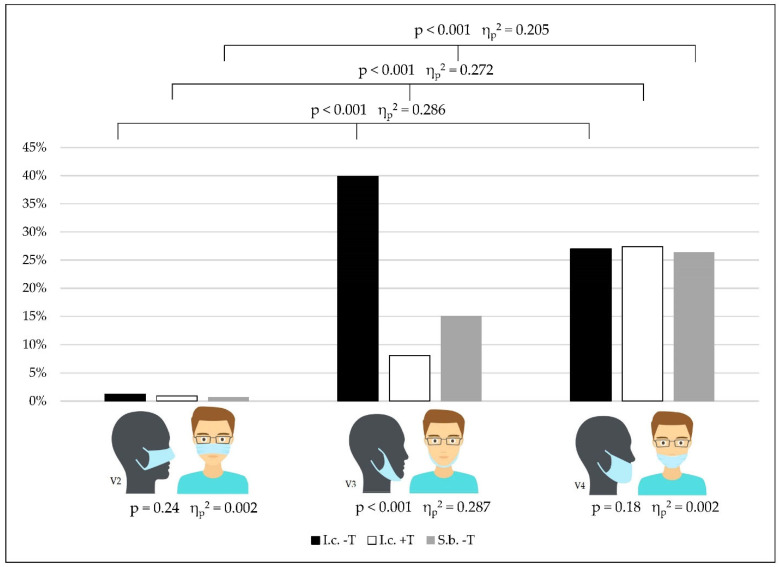
Percentages of self-estimated time of incorrect mask wearing (according to different situations in the schools and different variations in mask wearing).

We observed differences between the variations in incorrect face mask wearing in different situations in the schools among all the subgroups (*p* < 0.001) (Figure 4, Table 3 and Tables S8–S10). 

Students reported variant 3 (mouth and nose uncovered) as being dominant (main effect variants: *p* < 0.001, η_p_^2^ = 0.286) for the condition of being in class with no teacher present. Interaction effects between the situations in the schools and class membership (SiS*CM: *p* < 0.001, η_p_^2^ = 0.023) were found (Figure 4, Table 3, Tables S8 and S9).

In class with a teacher present or outside the classroom, students reported V4 (mouth covered, nose uncovered) as being dominant in total (I.c. +T: main effect variants: *p* < 0.001, η_p_^2^ = 0.272; S.b. -T: main effect variants: *p* < 0.001, η_p_^2^ = 0.205) in all of the subgroups (Figure 4, Table 3, Tables S8 and S9).

When comparing the results for variant 3 in the different situations at the schools, we found significant differences (V3: main effect for the different situations in school: *p* < 0.001, η_p_^2^ = 0.287) (Figure 4, Table 3, Tables S8 and S10).

The results of mask wearing for variants 2 and 4 did not differ between the different situations in the schools (V2: main effect: *p* = 0.24, η_p_^2^ = 0.002; V4: main effect: *p* = 0.18, η_p_^2^ = 0.002) (Figure 4, Table 3, Tables S8 and S10).

Additional information about the differences observed when face masks were worn incorrectly is reported in the Supplementary Materials.

## 4. Discussion

The results of our study show that face masks are frequently not worn correctly among children and adolescents. Additionally, it has become evident that the quality of face mask use is strongly dependent on the actual situations in classrooms and school buildings. In particular, the presence or absence of a teacher seems to play an important role in whether masks are worn correctly or not.

Thus far, very few studies have assessed the quality of face mask wearing during the COVID-19 pandemic. One study carried out in the USA reported a very high percentage (87.2%) of individuals wearing their face masks correctly in public places. The public place of the “school”, however, was not included in that study. The study reported an increased likelihood of incorrect face mask use among both children aged 2–11 years (OR 2.74) and adolescents aged 12–17 years (OR 1.36) compared with adults [24]. Our study is in line with a report from Zambia that states that only 59.3% of school children wore face masks correctly, a value that is also far below the target of 100% [25]. 

On average, in our study, students reported that, for less than 1/3 of the time spent without the presence of a teacher, face masks were worn correctly. The percentage of time spent with correct face mask use was significantly lower in classes with a sports focus when compared with the general branch classes (*p* < 0.001). Apparently, students in sports focus classes considered the mitigation measure of a “face mask” as less important than their counterparts in general classes. This may be due to the fact that students in sports focus classes were not obliged to wear face masks during sports lessons (mean: six hours per week). 

The crucial times of incorrect face mask use are, therefore, periods without a teacher present. Therefore, compliance could most likely be improved by the permanent presence of a teacher in secondary schools. However, in Austria, teachers usually change classrooms after each lesson, leaving students unattended in classrooms for a certain time. There are usually four to five such teacher changes per school day and class [26]. If this interval is assumed to be 3 to 5 min on average, the amount of time that students are in a classroom without a teacher accounts for 15 to 25 min of every day. Additionally, daily school breaks outside the classroom account for another 25 min [26].

Therefore, school children/students remain unattended for 40 to 55 min per school day. Based on the self-evaluation of this survey, many children/students “use” this time to uncover the openings of their airways (mostly both their noses and mouths). Our study did not evaluate the reasons for the children’s decision in doing so; however, it is likely that many children feel more comfortable without a face mask. Complaints and negative perceptions associated with face mask use might play a role too [27].

In the light of these findings, two questions arise:

(i) Is mandatory and correct face mask use effective in reducing the transmission of SARS-CoV-2 in classrooms and school buildings?
(ii) If so, what can be done to significantly improve the quality and compliance of face mask wearing?

These questions can be answered as follows:

Even if many school children wear their face masks incorrectly for a certain time, much time remains for which at least a limited protective effect can be assumed. Therefore, in critical epidemiological situations, obligations of face mask use in schools seem to be justified, even if compliance is (as shown in our study) limited. This obligation should, however, be supplemented with age-specific information for school children to increase their awareness and improve compliance.

Our study demonstrates that the quality of obligatory face mask use in schools is limited and may thus reduce the efficacy of this protective measure. The presence of teachers plays an important role in the rate of correct mask wearing, as unattended school children tend to uncover the openings of their airways. 

A limitation of our study is that we only had access to data from children in two schools in Austria (see Methods S2). In addition, the principal investigator was active at one study location, resulting in a higher participation rate at this location than at the second location (see Table S1). However, the sample size was large enough to enable an estimation of students’ habits at secondary schools in Austria.

Another limitation of our study is that the percentages reported for the different variations in the wearing of masks in the three situations in the schools resulted from the self-reflection of the participants, and no objective measurement was available.

## 5. Conclusions

To improve compliance, students should receive age-specific information about why and how they should wear a face mask correctly in order to reduce virus transmission in schools. In addition, students could spend breaks in the outdoor schoolyard or in the school building under increased teacher supervision. Improved coordination when switching teachers between different lessons should also be addressed in order to minimize the time spent without teacher supervision in the classroom as needed. The advantage of reduced transmission must, however, be carefully balanced against the negative side effects of mandatory face mask use in schools. 

Consideration should also be given to alternative means of reducing the need to wear face masks in classrooms. An option in this regard could be the effective ventilation and distribution of fresh air in the classroom, which has been shown to help reduce indoor virus transmission [28]. 

In the current situation, with the less pathogenic SARS-CoV-2 variants, the general long-term mandatory wearing of face masks in schools does not seem to be justified. However, more evidence is required and more studies on this issue are encouraged.

## Figures and Tables

**Table 1:** Detailed percentages of self-estimated correct mask wearing in different situations in the schools.

Variable	Situation at School		
	In Class without Teachers	In Class with Teacher	In School Building Outside the Classroom, without Teacher
M.S.-GB (*n* = 221)	45.8% ± 36.1%	70.4% ± 31.3%	65.6% ± 34.9%
M.S.-SF (*n* = 146)	28.9% ± 30.0%	62.1% ± 32.8%	55.1% ± 36.1%
H.S.-GB (*n* = 330)	30.6% ± 31.8%	65.9% ± 28.7%	58.0% ± 34.4%
H.S.-SF (*n* = 184)	19.7% ± 24.2%	52.7% ± 33.6%	50.7% ± 34.5%
♂ (*n* = 536)	30.9% ± 32.5%	61.2% ± 32.8%	56.5% ± 36.1%
♀ (*n* = 345)	33.4% ± 32.5%	67.5% ± 29.5%	60.1% ± 33.7%
All (*n* = 881)	31.9% ± 32.5%	63.7% ± 31.7%	57.9% ± 35.2%

Data are mean %. % = percentage. SiS = school situations. Correct face mask wearing = V1: mouth and nose are covered. Incorrect face mask wearing (sum of V2, V3, and V4) = V2: mouth is uncovered; V3: mouth and nose are uncovered; and V4: nose is uncovered. M = mean and SD = standard deviation. M.S. = middle school (students aged 12.8 ± 1.3 years old); H.S. = 4-year high school (students aged 16.7 ± 1.2 years old); GB = classes of a general school branch; and SF = school classes with a sports focus. ♂ = boys and ♀ = girls.

**Table 2:** ANOVA for correct mask wearing.

Effects	Variable	df	F	*p*-Value	η_p_^2^	Power
Between-subject effects	Sex	1	4.832	0.028	0.006	0.593
	C.M.	3	8.286	<0.001	0.028	0.993
	Sex*CM	3	6.368	<0.001	0.021	0.968
	Error	873				
Within-subject effects	SiS (I.c. -T–I.c. +T–S.b. -T)	1.950	356.023	<0.001	0.290	>0.99
	SiS*sex	1.950	1.456	0.23	0.002	0.309
	SiS*CM	5.849	4.235	<0.001	0.014	0.979
	SiS*sex*CM	5.849	3.819	0.001	0.013	0.964
	Error (SiS)	1702.032				

ANOVA = analysis of variance. df = degrees of freedom. F = test statistic. *p*-Value levels: significant = *p* < 0.05; very significant = *p* < 0.01; and highly significant = *p* < 0.001. η_p_^2^ = partial eta square. Sex: ♂ = boys and ♀ = girls. CM = class membership (M.S. GB, M.S. SF, H.S. GB, and H.S. SF). I.c. -T = in classes without teachers; I.c. +T = in classes with teachers; and S.b. -T = in school buildings outside class and without teachers. M = mean and SD = standard deviation. M.S. = middle school (students aged 12.8 ± 1.3 years old); H.S. = 4-year high school (students aged 16.7 ± 1.2 years old); GB = classes of a general school branch; and SF = school classes with a sports focus.

**Table 3:** ANOVAs for incorrect mask wearing (situations in the schools and mask wearing variants).

Situation in School	Effect	Variable	df	F	*p*-Value	η_p_^2^	Power
I.c. -T	Between-subject effects	Sex	1	0.786	0.38	0.001	0.143
		CM	3	16.247	<0.001	0.053	>0.99
		Sex*CM	3	3.192	0.023	0.011	0.739
		Error	873				
	Within-subject effects	Variant (V2–V3–V4)	1.322	350.416	<0.001	0.286	0.99
		Variant*sex	1.322	1.660	0.20	0.002	0.285
		Variant*CM	3.965	6.986	<0.001	0.023	0.995
		Variant*sex*CM	3.965	0.910	0.46	0.003	0.290
		Error	1153.931				
I.c. +T	Between-subject effects	Sex	1	7.311	0.007	0.008	0.771
		CM	3	2.797	0.039	0.010	0.675
		Sex*CM	3	9.210	<0.001	0.031	0.997
		Error	873				
	Within-subject effects	Variant (V2–V3–V4)	1.449	326.195	<0.001	0.272	0.99
		Variant*sex	1.449	1.258	0.28	0.001	0.236
		Variant*CM	4.347	2.140	0.07	0.007	0.664
		Variant*sex*CM	4.347	5.056	<0.001	0.017	0.974
		Error	1265.078				
s.b. -T	Between-subject effects	Sex	1	3.163	0.08	0.004	0.427
		CM	3	2.542	0.06	0.009	0.629
		Sex*CM	3	4.447	0.004	0.015	0.878
		Error	873				
	Within-subject effects	Variant (V2–V3–V4)	1.552	224.885	<0.001	0.205	0.99
		Variant*sex	1.552	1.228	0.29	0.001	0.238
		Variant*CM	4.657	0.880	0.49	0.003	0.307
		Variant*sex*CM	4.657	2.547	0.030	0.009	0.772
		Error	1355.299				
**Mask Wearing Variant**	**Effect**	**Variable**	**df**	**F**	***p*-Value**	**η_p_^2^**	**Power**
V2	Between-subject effects	Sex	1	3.443	0.06	0.004	0.458
		CM	3	0.186	0.91	0.001	0.085
		Sex*CM	3	0.548	0.65	0.002	0.164
		Error	873				
	Within-subject effects	SiS (I.c. -T–I.c. +T–S.b. -T)	1.902	1.443	0.24	0.002	0.302
		SiS*sex	1.902	1.865	0.16	0.002	0.380
		SiS*CM	5.707	1.350	0.23	0.005	0.520
		SiS*sex*CM	5.707	1.370	0.23	0.005	0.527
		Error	1660.843				
V3	Between-subject effects	Sex	1	2.324	0.13	0.003	0.331
		CM	3	7.102	<0.001	0.024	0.982
		Sex*CM	3	1.512	0.21	0.005	0.401
		Error	873				
	Within-subject effects	SiS (I.c. -T–I.c. +T–S.b. -T)	1.627	351.626	<0.001	0.287	0.99
		SiS*sex	1.627	0.402	0.63	0.001	0.109
		SiS*CM	4.881	7.953	<0.001	0.027	0.99
		SiS*sex*CM	4.881	1.271	0.27	0.004	0.450
		Error	1420.360				
V4	Between-subject effects	Sex	1	1.580	0.21	0.002	0.241
		CM	3	4.243	0.005	0.014	0.861
		Sex*CM	3	6.262	<0.001	0.021	0.966
		Error	873				
	Within-subject effects	SiS (I.c. -T–I.c. +T–S.b. -T)	1.946	1.730	0.18	0.002	0.360
		SiS*sex	1.946	3.818	0.023	0.004	0.687
		SiS*CM	5.839	0.546	0.77	0.002	0.219
		SiS*sex*CM	5.839	3.481	0.002	0.012	0.945
		Error	1699.157				

ANOVA = analysis of variance. df = degrees of freedom. F = test statistic. *p*-Value levels: significant = *p* < 0.05; very significant = *p* < 0.01; and highly significant = *p* < 0.001. η_p_^2^ = partial eta square. Sex: ♂ = boys and ♀ = girls. CM = class membership (M.S. GB, M.S. SF, H.S. GB, and H.S. SF). M.S. = middle school (students aged 12.8 ± 1.3 years old); H.S. = 4-year high school (students aged 16.7 ± 1.2 years old); GB = classes of a general school branch; and SF = school classes with a sports focus. SiS = school situations. I.c. -T = in classes without teachers; I.c. +T = in classes with teachers; and S.b. -T = in school buildings outside class and without teachers. V2 = mouth is uncovered; V3 = mouth and nose are uncovered; and V4 = nose is uncovered.

## Data Availability

The data presented in this study are available on request from the corresponding author. The data are not publicly available due to privacy/ethical restrictions.

## Supplementary Materials

The following are available online at https://www.mdpi.com/article/10.3390/healthcare10091641/s1, Methods S1. Information about the questionnaire format, Methods S2: Additional information about the selection of the study participants, Table S1. Overview of the study flow, Table S2. Sample characteristics for the total study population and the middle and high school subgroups, Table S3. Differences in correct mask wearing between situations in the schools for the total population and all subgroups, Table S4. Additional information of the percentage of self-estimated correct mask wearing in different situations in the schools, Table S5. Post hoc tests for correct mask wearing in different situations in the schools based on the estimated marginal means, Table S6. Post hoc tests for correct mask wearing between class memberships based on the estimated marginal means, Table S7. Post hoc tests for correct mask wearing between class memberships for different situations in the schools based on the observed means, Table S8. Descriptive information of percentages of self-estimated incorrect mask wearing in different situations in the schools, Table S9. Post hoc tests for incorrect mask wearing in different situations in the schools between class memberships based on the estimated marginal means, Table S10. Post hoc tests for incorrect mask wearing in different variants of mask wearing between class memberships based on the estimated marginal means.
