# Supplementary material for: How Middle and High School Students Wear Their Face Masks in Classrooms and School Buildings
Source: Healthcare (Basel). 2022 Aug 28;10(9):1641. doi: 10.3390/healthcare10091641 (PMC9498795; doi:10.3390/healthcare10091641)
Supplement: Supplementary file 1 [file healthcare-10-01641-s001.zip › healthcare-1874958-supplementary.pdf]

Supplementary material

How Middle and High School Students Wear Their Face Masks in Classrooms and School Buildings.

Gerald Jarnig, Reinhold Kerbl, Mireille N.M. van Poppel

This supplementary material has been provided by the authors to give readers additional information about their work.

Methods S1. Information about the questionnaire format

Methods S2: Additional information about the selection of the study participants

Table S1. Overview of the study flow

Table S2. Sample characteristics for the total study population and the middle and high school subgroups

Table S3. Differences in correct mask wearing between situations in the schools for total population and all subgroups.

Table S4. Additional information of the percentage of self-estimated correct mask wearing in different situation in the schools

Table S5. Post hoc tests for correct mask wearing in different situations in the schools based on the estimated marginal means

Table S6. Post hoc tests for correct mask wearing between class membership based on the estimated marginal means

Table S7. Post hoc tests for correct mask wearing between class membership for different situations in the schools based on observed means

Table S8. Descriptive information of percentage of self-estimated incorrect mask wearing in different situations in the schools

Table S9. Post hoc tests for incorrect mask wearing in different situations in the schools between class membership based on the estimated marginal means

Table S10. Post hoc tests for incorrect mask wearing in different variants of mask wearing between class membership based on the estimated marginal means

Methods S1. Information about the questionnaire format

The following variants were asked for each of the three different situations:

Variant 1 (V1) = mouth and nose covered
Variant 2 (V2) = mouth uncovered; nose covered
Variant 3 (V3) = mouth and nose uncovered
Variant 4 (V4) = mouth covered; nose uncovered

V1 shows correct wearing of face mask. V2 to V4 demonstrate an incorrect wearing of face mask.

The following questionnaire was answered by the study participants:

Study questionnaire: Mask wearing in different situations at school

Introduction: (was additionally given in words)

How did you personally wear the face mask at school in different situations? Please estimate the percentage for each variant and in each school situation!
(Please note that the sum of the 4 variants should be 100% for each school situation, you also have the option of assigning a self-chosen percentage for the individual variants).

Situation 1: **In class**, when **no teacher** was present?

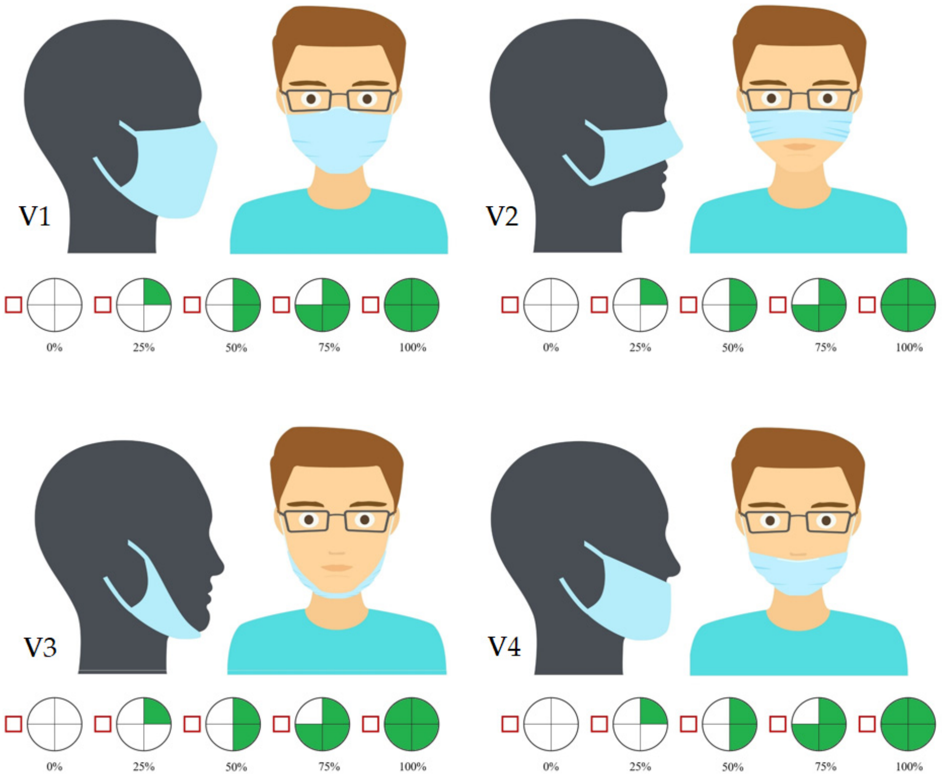

Image material purchased - ©Adobe Stock

Situation 2: **In class**, when **teacher was present**?

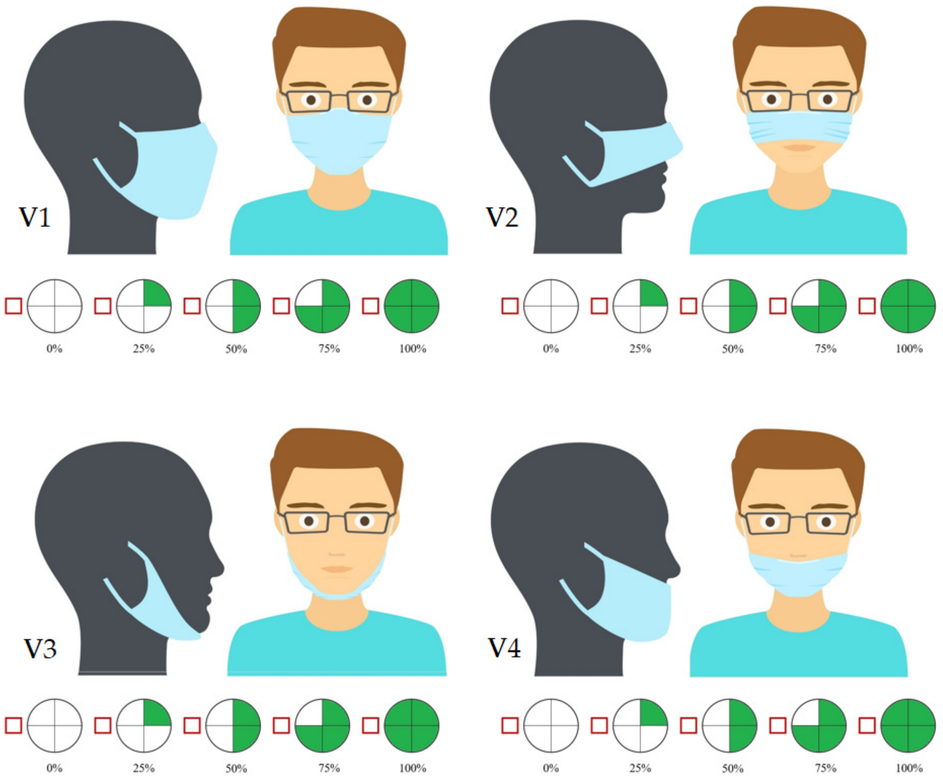

Image material purchased - ©Adobe Stock

Situation 3: In **school building outside classroom** and **no teachers** present?

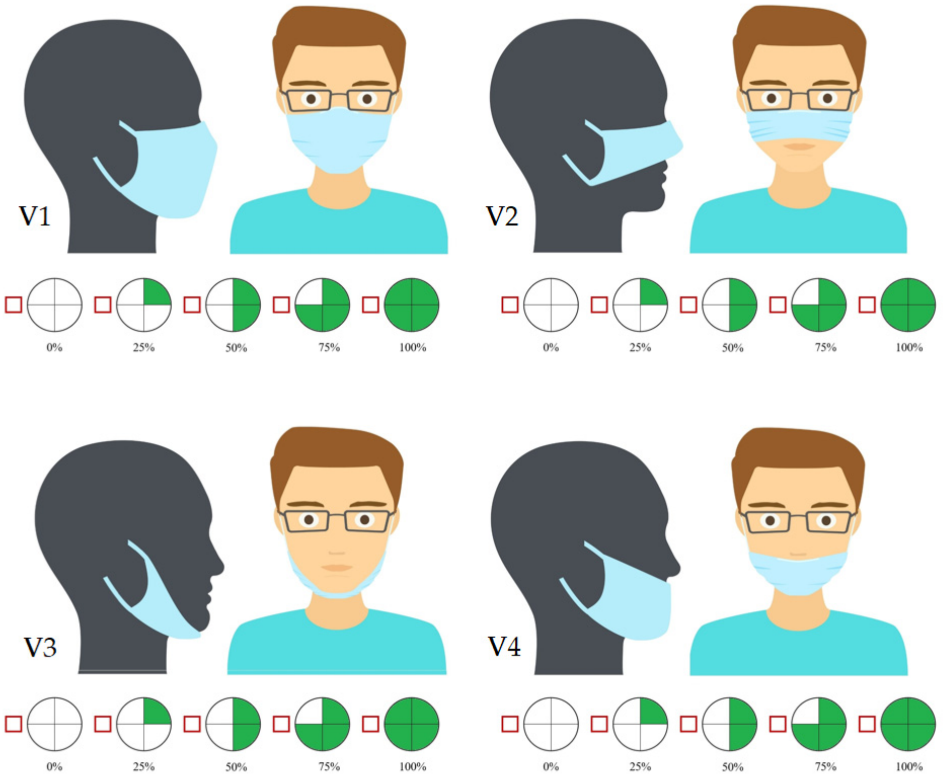

Image material purchased - ©Adobe Stock

Methods S2: Additional information about the selection of the study participants

Due to the COVID-19 pandemic, the participation in Austrian school sports was strictly restricted in the school years 2020/21 and 2021/22. There were very strict guidelines that disallowed "normal" and conventional sports classes and, in some cases, led to the complete elimination of sports activities in schools.
Based on the activity of the study director in the area of the middle school (BRG/BORG practically middle school of the PH Carinthia) with a sports focus (M.S. SF) and the pilot school classes located there with the focus "middle school for development in competitive sports", he had the opportunity to observe 4 school classes in the current school year 2021/22, in which the practice of school sports in school was as well as possible without restrictions.
The general status of "top sport" applied to the classes described, thus enabling the practice of exercise and sport in compliance with the generally current guidelines and safety measures of top sport. The same applied at this school location also for high school classes (H.S. SF), which are trained in sports within the framework of the School Sports Performance Model Carinthia (SSLK).
In parallel, middle (M.S. GB) and high school (H.S. GB) classes were also located at this school campus, which had a general school branch (GB), the practice of physical activities and sports was only allowed under strict restrictions here.
Both school branches had strict COVID-19 mitigation measures during cognitive lessons, which were relaxed for classes with SF during sports lessons (no face mask or other restrictions).
There was a second school campus in Austria in the province of Salzburg where comparable conditions existed (Christian-Doppler-Gymnasium, 5020 Salzburg).
On the initiative of the study director, a study was designed together with the Austrian Pediatrics and Adolescent Medicine, which was further commissioned and funded by the Ministry of Education, Science and Research.
Due to the activity of the study director in the area of the middle school (Carinthia), a very high study participation could be made possible through repeated inquiries and reminding.
At the second school campus (Salzburg), personal contact was lacking and thus only a significantly lower percentage of complete data could be collected.
Therefore, the first study aim (The impact of wearing FFP2 masks in classrooms on SARS-CoV-2 infection rates) could only be investigated at the school campus in Carinthia.
For the second study aim (results reported in the present study), data from both school locations were included.
Due to the large difference in the percentage of study participants, the different school location membership, was not taken into account in the descriptive reporting and all statistical analyses used.

Table S1. Overview of the study flow

Overview of the study invitation									
**Variable**	**C.M.**	**T.S.**	**I**	**C.P.**	**D**	**E.R.**	**N.R.**	**C.D.**	**Q.n.C.**
Carinthia	M.S. GB 1	24	24	24	0	0	0	14	10
	M.S. GB 2	24	24	24	0	0	0	23	1
	M.S. GB 3	20	20	20	0	0	0	16	4
	M.S. GB 4	25	25	25	0	0	0	9	16
	M.S. GB 5	23	23	23	0	0	0	21	2
	M.S. GB 6	25	25	25	0	0	0	19	6
	M.S. GB 7	24	24	24	0	0	0	18	6
	M.S. GB 8	26	26	25	1	0	0	21	4
	M.S. SF 1	26	26	26	0	0	0	24	2
	M.S. SF 2	24	24	24	0	0	0	22	2
	M.S. SF 3	25	24	24	0	0	0	23	1
	M.S. SF 4	25	25	25	0	0	0	24	1
	H.S. GB 1	28	28	27	1	0	0	23	4
	H.S. GB 2	31	31	31	0	0	0	31	0
	H.S. GB 3	31	29	29	0	0	0	29	0
	H.S. GB 4	21	19	19	0	0	0	19	0
	H.S. GB 5	24	24	24	0	0	0	24	0
	H.S. GB 6	31	30	30	0	0	0	27	3
	H.S. GB 7	18	18	18	0	0	0	16	2
	H.S. GB 8	24	22	22	0	0	0	22	0
	H.S. GB 9	30	30	30	0	0	0	29	1
	H.S. SF 1	27	27	27	0	0	0	27	0
	H.S. SF 2	26	25	25	0	0	0	23	2
	H.S. SF 3	27	24	24	0	0	0	21	3
	H.S. SF 4	20	20	20	0	0	0	20	0
Total Carinthia	25 classes	629	617	615	2	0	0	545	70
		100.0%	100.0%	99.7%	0.3%	0.0%	0.0%	88.3%	11.3%
Salzburg	M.S. GB 9	23	23	6	0	0	17	6	0
	M.S. GB 10	24	24	9	0	2	13	9	0
	M.S. GB 11	26	26	12	0	0	14	11	1
	M.S. GB 12	26	26	0	0	0	26	0	0
	M.S. GB 13	26	26	14	0	0	12	13	1
	M.S. GB 14	23	23	4	0	1	18	4	0
	M.S. GB 15	22	22	11	0	1	10	10	1
	M.S. GB 16	29	29	7	0	0	22	6	1
	M.S. GB 17	28	28	0	0	0	28	0	0
	M.S. GB 18	28	28	10	0	0	18	10	0
	M.S. GB 19	26	26	4	0	0	22	4	0
	M.S. GB 20	26	26	8	0	0	18	7	1
	M.S. SF 5	25	25	10	0	0	15	10	0
	M.S. SF 6	25	25	11	0	0	14	9	2
	M.S. SF 7	28	28	11	0	0	17	11	0
	M.S. SF 8	30	30	19	0	0	11	16	3
	M.S. SF 9	25	25	5	0	2	18	5	0
	M.S. SF 10	24	24	0	0	0	24	0	0
	M.S. SF 11	28	28	2	0	0	26	2	0

	H.S. GB 9	27	27	18	0	1	8	17	1
	H.S. GB 10	28	28	25	0	3	0	25	0
	H.S. GB 11	10	10	0	0	0	10	0	0
	H.S. GB 12	16	16	9	0	0	7	9	0
	H.S. GB 13	20	20	16	0	0	4	16	0
	H.S. GB 14	21	21	19	0	0	2	18	1
	H.S. GB 15	27	27	16	0	11	0	16	0
	H.S. GB 16	17	17	9	0	0	8	9	0
	H.S. SF 5	27	27	21	3	1	2	21	0
	H.S. SF 6	16	16	13	0	0	3	13	0
	H.S. SF 7	23	23	19	0	3	1	17	2
	H.S. SF 8	18	18	14	0	0	4	14	0
	H.S. SF 9	21	21	18	0	0	3	17	1
	H.S. SF 10	20	20	11	0	0	9	11	0
Total Salzburg	33 classes	783	783	351	3	25	404	336	15
		100.0%	100.0%	44.8%	0.4%	3.2%	51.6%	42.9%	1.9%
All	58 classes	1412	1400	966	5	25	404	881	85
		100.0%	100.0%	69.0%	0.4%	1.8%	28.9%	62.9%	6.1%

C.M.= class membership, T.S. = total students, I = invited, C.P. = consented to participate, D = declined, E.R. = empty response, N.R. = no response, C.D. = complete data, Q.n.C. = Questionnaire was not filled completely, M.S. = middle school (students aged 12.8 ± 1.3 years old); H.S. = 4-year high school (students aged 16.7 ± 1.2 years old); GB = classes with a general school branch; and SF = school classes with a sports focus.

Table S2. Sample characteristics for the total study population and the middle and high school subgroups

Variable	All (n = 881)	GB (n = 551)	SF (n = 330)	X^2	t	*P-Value*	p-lvl
female, No. (%)	265 (43.2%)	272 (49.4%)	73 (22.1%)	64.296		<.001	***
Age at index, mean (SD)	15.1 ± 2.3	15.3 ± 2.3	14.7 ± 2.2		3.908	<.001	***
	M.S.						
	All (n = 367)	GB (n = 221)	SF (n = 146)				
female	140 (38.2%)	100 (45.2%)	40 (27.4%)	11.874		0.001	**
Age at index	12.8 ± 1.3	13.0 ± 1.3	12.6 ± 1.2		3.037	0.003	**
	H.S.						
	All (n = 514)	GB (n = 330)	SF (n = 184)				
female	154 (39.9%)	172 (52.1%)	33 (17.9%)	57.582		<.001	***
Age at index	16.7 ± 1.2	16.9 ± 1.2	16.4 ± 1.2		4.477	<.001	***

Data are absolute numbers (No.) and percentages (%), X^2 = Chi-Square Test value, t = test statistic t-test, P-value = levels (p-lvl): * = p < 0.05; ** = p < 0.01; and *** = p < 0.001. No. = Number, % = Percentage. I.c. -T = in classes without teachers; I.c. +T = in classes with teachers; and S.b. -T = in school buildings outside classes and without teachers. M = mean and SD = standard deviation. M.S. = middle school (students aged 12.8 ± 1.3 years old); H.S. = 4-year high school (students aged 16.7 ± 1.2 years old); GB = classes with a general school branch; and SF = school classes with a sports focus. Sex = ♂ = boys and ♀ = girls.

Table S3. Differences in correct mask wearing between situations in the schools for total population and all subgroups.

Groups	SiS		t	*P*-Value	p-lvl
	**I.c. –T (No Teacher)**	**I.c. +T (Teacher Present)**			
M.S. - GB (n = 221)	45.8% ± 36.1%	70.4% ± 31.3%	-11.563	<.001	***
M.S. - SF (n = 146)	28.9% ± 30.0%	62.1% ± 32.8%	-11.483	<.001	***
H.S. - GB(n = 330)	30.6% ± 31.8%	65.9% ± 28.7%	-20.614	<.001	***
H.S. - SF (n = 184)	19.7% ± 24.2%	52.7% ± 33.6%	-15.056	<.001	***
♂ (n = 536)	30.9% ± 32.5%	61.2% ± 32.8%	-22.235	<.001	***
♀ (n = 345)	33.4% ± 32.5%	67.5% ± 29.5%	-19.641	<.001	***
All (n = 881)	31.9% ± 32.5%	63.7% ± 31.7%	-29.615	<.001	***
	**SiS**				
	**I.c. –T (No Teacher)**	**S.b. -T**			
M.S. - GB (n = 221)	45.8% ± 36.1%	65.6% ± 34.9%	-8.733	<.001	***
M.S. - SF (n = 146)	28.9% ± 30.0%	55.1% ± 36.1%	-8.363	<.001	***
H.S. - GB(n = 330)	30.6% ± 31.8%	58.0% ± 34.4%	-14.046	<.001	***
H.S. - SF (n = 184)	19.7% ± 24.2%	50.7% ± 34.5%	-12.307	<.001	***
♂ (n = 536)	30.9% ± 32.5%	56.5% ± 36.1%	-16.983	<.001	***
♀ (n = 345)	33.4% ± 32.5%	60.1% ± 33.7%	-13.787	<.001	***
All (n = 881)	31.9% ± 32.5%	57.9% ± 35.2%	-21.884	<.001	***
	**SiS**				
	**I.c. +T (Teacher Present)**	**S.b. -T**			
M.S. - GB (n = 221)	70.4% ± 31.3%	65.6% ± 34.9%	2.240	0.026	*
M.S. - SF (n = 146)	62.1% ± 32.8%	55.1% ± 36.1%	2.201	0.029	*
H.S. - GB(n = 330)	65.9% ± 28.7%	58.0% ± 34.4%	5.080	<.001	***
H.S. - SF (n = 184)	52.7% ± 33.6%	50.7% ± 34.5%	0.873	0.38	
♂ (n = 536)	61.2% ± 32.8%	56.5% ± 36.1%	3.305	0.001	**
♀ (n = 345)	67.5% ± 29.5%	60.1% ± 33.7%	4.581	<.001	***
All (n = 881)	63.7% ± 31.7%	57.9% ± 35.2%	5.376	<.001	***

Data are absolute numbers (No.) and percentages (%), t = test statistic t-test, P-value = levels (p-lvl): * = $p < 0.05$; ** = $p < 0.01$; and *** = $p < 0.001$. No. = Number, % = Percentage. SiS = school situations. I.c. -T = in classes without teachers; I.c. +T = in classes with teachers; and S.b. -T = in school buildings outside classes and without teachers. M = mean and SD = standard deviation. M.S. = middle school (students aged 12.8 ± 1.3 years old); H.S. = 4-year high school (students aged 16.7 ± 1.2 years old); GB = classes with a general school branch; and SF = school classes with a sports focus. Sex = ♂ = boys and ♀ = girls.

Table S4. Additional information of the percentage of self-estimated correct mask wearing in different situation in the schools

CM	Category	I.c. -T			I.c. +T			S.b. -T		
		M ± SD			M ± SD			M ± SD		
M.S. - GB	♂ (n = 121)	49.33%	±	36.98%	71.14%	±	32.96%	69.75%	±	35.49%
	♀ (n = 100)	41.60%	±	34.66%	69.55%	±	29.32%	60.62%	±	33.78%
	All (n = 221)	45.83%	±	36.08%	70.42%	±	31.31%	65.62%	±	34.94%
M.S. - SF	♂ (n = 106)	26.01%	±	29.17%	63.34%	±	34.60%	52.33%	±	37.27%
	♀ (n = 40)	36.52%	±	31.14%	58.87%	±	27.49%	62.54%	±	31.90%
	All (n = 146)	28.89%	±	29.98%	62.12%	±	32.78%	55.13%	±	36.06%
H.S. - GB	♂ (n = 158)	32.16%	±	32.51%	65.63%	±	26.64%	57.83%	±	34.29%
	♀ (n = 172)	29.22%	±	31.25%	66.16%	±	30.54%	58.24%	±	34.67%
	All (n = 330)	30.63%	±	31.85%	65.91%	±	28.69%	58.04%	±	34.44%
H.S. - SF	♂ (n = 151)	18.09%	±	22.58%	47.01%	±	32.85%	47.48%	±	34.52%
	♀ (n = 33)	27.06%	±	29.93%	79.00%	±	23.38%	65.66%	±	30.88%
	All (n = 184)	19.70%	±	24.22%	52.75%	±	33.64%	50.74%	±	34.53%

Data are absolute numbers (No.) and percentages (%), No. = Number, % = Percentage. CM = class membership (M.S. GB, M.S. SF, H.S. GB, and H.S. SF). I.c. -T = in classes without teachers; I.c. +T = in classes with teachers; and S.b. -T = in school buildings outside classes and without teachers. M = mean and SD = standard deviation. M.S. = middle school (students aged 12.8 ± 1.3 years old); H.S. = 4-year high school (students aged 16.7 ± 1.2 years old); GB = classes with a general school branch; and SF = school classes with a sports focus. Sex = ♂ = boys and ♀ = girls.

Table S5. Post hoc tests for correct mask wearing in different situations in the schools based on the estimated marginal means

Pairwise comparison			Mean diff	95% CI		SE	*P*-Value [a]	p-lvl
				LB	UB			
I.c. -T	vs.	I.c. +T	-32.59%	-35.58%	-29.60%	1.247	<.001	***
I.c. -T	vs.	S.b. -T	-26.81%	-30.18%	-23.44%	1.404	<.001	***
I.c. +T	vs.	S.b. -T	5.79%	2.78%	8.79%	1.253	<.001	***

a = adjusted for multiple comparisons using Bonferroni correction.

Mean diff = mean difference based on the estimated marginal means, CI = confidence interval, LB = lower bound, UB = upper bound, SE = standard error. P-Value = levels (p-lvl): * = $p < 0.05$; ** = $p < 0.01$; and *** = $p < 0.001$. I.c. -T = in classes without teachers; I.c. +T = in classes with teachers; and S.b. -T = in school buildings outside classes and without teachers.

Table S6. Post hoc tests for correct mask wearing between class membership based on the estimated marginal means

Pairwise comparison			Mean diff	95% CI		SE	*P*-Value [a]	p-lvl
				LB	UB			
M.S. GB	vs.	M.S. SF	10.40%	2.47%	18.32%	2.997	.003	**
M.S. GB	vs.	H.S. GB	8.79%	2.77%	14.81%	2.277	<.001	***
M.S. GB	vs.	H.S. SF	12.95%	4.84%	21.06%	3.067	<.001	***
M.S. SF	vs.	H.S. GB	-1.60%	-9.05%	5.85%	2.818	>.99	
M.S. SF	vs.	H.S. SF	2.55%	-6.67%	11.78%	3.488	>.99	
H.S. GB	vs.	H.S. SF	4.16%	-3.49%	11.80%	2.892	.91	

a = adjusted for multiple comparisons using Bonferroni correction.

Mean diff = mean difference based on the estimated marginal means, CI = confidence interval, LB = lower bound, UB = upper bound, SE = standard error. P-Value = levels (p-lvl): * = $p < 0.05$; ** = $p < 0.01$; and *** = $p < 0.001$. M.S. = middle school (students aged 12.8 ± 1.3 years old); H.S. = 4-year high school (students aged 16.7 ± 1.2 years old); GB = classes with a general school branch; and SF = school classes with a sports focus.

Table S7. Post hoc tests for correct mask wearing between class membership for different situations in the schools based on observed means

School situations	Pairwise comparison			Mean diff	95% CI		SE	*P*-Value [a]	p-lvl
					LB	LB			
I.c. -T	M.S. GB	vs.	M.S. SF	16.94%	8.15%	25.73%	3.325	<.001	***
	M.S. GB	vs.	H.S. GB	15.20%	8.03%	22.36%	2.710	<.001	***
	M.S. GB	vs.	H.S. SF	26.13%	17.91%	34.36%	3.111	<.001	***
	M.S. SF	vs.	H.S. GB	-1.74%	-9.93%	6.45%	3.098	>.99	
	M.S. SF	vs.	H.S. SF	9.19%	0.06%	18.33%	3.455	.048	*
	H.S. GB	vs.	H.S. SF	10.93%	3.35%	18.52%	2.868	.001	**
I.c. +T	M.S. GB	vs.	M.S. SF	8.30%	-0.35%	16.95%	3.270	.07	
	M.S. GB	vs.	H.S. GB	4.52%	-2.53%	11.56%	2.665	.54	
	M.S. GB	vs.	H.S. SF	17.67%	9.58%	25.77%	3.060	<.001	***
	M.S. SF	vs.	H.S. GB	-3.79%	-11.85%	4.27%	3.048	>.99	
	M.S. SF	vs.	H.S. SF	9.37%	0.38%	18.36%	3.399	.036	*
	H.S. GB	vs.	H.S. SF	13.16%	5.70%	20.62%	2.821	<.001	***
S.b. -T	M.S. GB	vs.	M.S. SF	10.49%	0.72%	20.27%	3.697	.028	*
	M.S. GB	vs.	H.S. GB	7.58%	-0.39%	15.55%	3.013	.07	
	M.S. GB	vs.	H.S. SF	14.88%	5.73%	24.03%	3.459	<.001	***
	M.S. SF	vs.	H.S. GB	-2.91%	-12.03%	6.20%	3.445	>.99	
	M.S. SF	vs.	H.S. SF	4.39%	-5.77%	14.55%	3.842	>.99	
	H.S. GB	vs.	H.S. SF	7.30%	-1.13%	15.74%	3.189	.13	

a = adjusted for multiple comparisons using Bonferroni correction.

Mean diff = mean difference based on observed means, CI = confidence interval, LB = lower bound, UB = upper bound, SE = standard error, P-value = levels (p-lvl): * = $p < 0.05$; ** = $p < 0.01$; and *** = $p < 0.001$. No. = Number, % = Percentage. I.c. -T = in classes without teachers; I.c. +T = in classes with teachers; and S.b. -T = in school buildings outside classes and without teachers. M = mean and SD = standard deviation. M.S. = middle school (students aged 12.8 ± 1.3 years old); H.S. = 4-year high school (students aged 16.7 ± 1.2 years old); GB = classes with a general school branch; and SF = school classes with a sports focus.

Table S8. Descriptive information of percentage of self-estimated incorrect mask wearing in different situations in the schools

Variable	I.c. -T			I.c. +T			S.b. -T		
	V2	V3	V4	V2	V3	V4	V2	V3	V4
M.S. - GB (n = 221), M ± SD	1.6% ± 8.5%	28.8% ± 31.7%	23.8% ± 26.2%	1.1% ± 8.1%	7.1% ± 17.3%	21.3% ± 25.5%	0.4% ± 2.5%	12.3% ± 23.7%	21.7% ± 25.8%
M.S. - SF (n = 146), M ± SD	1.6% ± 6.3%	37.2% ± 32.1%	32.3% ± 25.6%	0.5% ± 3.6%	9.6% ± 18.5%	27.7% ± 25.6%	0.9% ± 4.8%	14.1% ± 21.5%	29.9% ± 29.5%
H.S. - GB(n = 330) , M ± SD	0.9% ± 5.2%	42.5% ± 31.4%	26.0% ± 23.1%	0.7% ± 4.2%	7.2% ± 13.4%	26.2% ± 24.8%	0.5% ± 3.6%	15.8% ± 22.4%	25.6% ± 24.5%
H.S. - SF (n = 184) , M ± SD	1.2% ± 5.5%	50.9% ± 32.9%	28.3% ± 25.3%	1.2% ± 5.7%	9.6% ± 17.2%	36.4% ± 31.0%	1.0% ± 4.6%	17.7% ± 25.4%	30.6% ± 28.4%
♂ (n = 536) , M ± SD	1.5% ± 7.2%	41.2% ± 33.7%	26.5% ± 25.0%	1.2% ± 6.8%	8.9% ± 17.5%	28.7% ± 28.0%	0.8% ± 4.2%	15.5% ± 25.0%	27.2% ± 27.8%
♀ (n = 345) , M ± SD	0.9% ± 4.9%	38.0% ± 31.2%	27.7% ± 24.8%	0.4% ± 3.0%	6.8% ± 13.9%	25.2% ± 25.1%	0.5% ± 3.2%	14.3% ± 20.4%	25.1% ± 25.0%
All (n = 881) , M ± SD	1.2% ± 6.4%	39.9% ± 32.8%	27.0% ± 24.9%	0.9% ± 5.7%	8.1% ± 16.2%	27.4% ± 27.0%	0.7% ± 3.8%	15.1% ± 23.3%	26.4% ± 26.7%

Data are absolute numbers (No.) and percentages (%), No. = Number, % = Percentage, I.c. -T = in classes without teachers, M = mean and SD = standard deviation. Sex = ♂ = boys and ♀ = girls. M.S. = middle school (students aged 12.8 ± 1.3 years old); H.S. = 4-year high school (students aged 16.7 ± 1.2 years old); GB = classes with a general school branch; and SF = school classes with a sports focus. I.c. -T = in classes without teachers; I.c. +T = in classes with teachers; and S.b. -T = in school buildings outside classes and without teachers. V2 = mouth is uncovered; V3 = mouth and nose are uncovered; and V4 = nose is uncovered.

Table S9. Post hoc tests for incorrect mask wearing in different situations in the schools between class membership based on the estimated marginal means

Variable	Pairwise comparison			Mean diff	95% CI		SE	*P*-Value [a]	p-lvl
					LB	LB			
I.c. -T	M.S. GB	vs.	M.S. SF	-4.73%	-7.89%	-1.58%	1.193	<.001	***
	M.S. GB	vs.	H.S. GB	-4.92%	-7.32%	-2.53%	0.906	<.001	***
	M.S. GB	vs.	H.S. SF	-7.63%	-10.86%	-4.40%	1.221	<.001	***
	M.S. SF	vs.	H.S. GB	-0.19%	-3.16%	2.77%	1.121	>.99	
	M.S. SF	vs.	H.S. SF	-2.90%	-6.57%	0.77%	1.388	.22	
	H.S. GB	vs.	H.S. SF	-2.71%	-5.75%	0.34%	1.151	.11	
I.c. +T	M.S. GB	vs.	M.S. SF	-3.08%	-6.18%	0.02%	1.173	.05	
	M.S. GB	vs.	H.S. GB	-1.48%	-3.84%	0.87%	0.891	.58	
	M.S. GB	vs.	H.S. SF	-2.45%	-5.62%	0.73%	1.201	.25	
	M.S. SF	vs.	H.S. GB	1.60%	-1.32%	4.51%	1.103	.89	
	M.S. SF	vs.	H.S. SF	0.63%	-2.98%	4.24%	1.365	>.99	
	H.S. GB	vs.	H.S. SF	-0.96%	-3.96%	2.03%	1.132	>.99	
outC_woT	M.S. GB	vs.	M.S. SF	-2.58%	-6.09%	0.92%	1.326	.31	
	M.S. GB	vs.	H.S. GB	-2.38%	-5.05%	0.28%	1.007	.11	
	M.S. GB	vs.	H.S. SF	-2.87%	-6.46%	0.72%	1.357	.21	
	M.S. SF	vs.	H.S. GB	0.20%	-3.10%	3.50%	1.247	>.99	
	M.S. SF	vs.	H.S. SF	-0.29%	-4.37%	3.79%	1.543	>.99	
	H.S. GB	vs.	H.S. SF	-0.49%	-3.87%	2.90%	1.280	>.99	

a = adjusted for multiple comparisons using Bonferroni correction.
Mean diff = mean difference based on the estimated marginal means, CI = confidence interval, LB = lower bound, UB = upper bound, SE = standard error, P-value = levels (p-lvl): * = $p < 0.05$; ** = $p < 0.01$; and *** = $p < 0.001$. No. = Number, % = Percentage. I.c. -T = in classes without teachers; I.c. +T = in classes with teachers; and S.b. -T = in school buildings outside classes and without teachers. M.S. = middle school (students aged 12.8 ± 1.3 years old); H.S. = 4-year high school (students aged 16.7 ± 1.2 years old); GB = classes with a general school branch; and SF = school classes with a sports focus.

Table S10. Post hoc tests for incorrect mask wearing in different variants of mask wearing between class membership based on the estimated marginal means

Variable	Pairwise comparison			Mean diff	95% CI		SE	*P*-Value [a]	p-lvl
					LB	LB			
V2	M.S. GB	vs.	M.S. SF	0.19%	-1.10%	1.48%	0.489	>.99	
	M.S. GB	vs.	H.S. GB	0.24%	-0.74%	1.23%	0.371	>.99	
	M.S. GB	vs.	H.S. SF	0.00%	-1.32%	1.33%	0.500	>.99	
	M.S. SF	vs.	H.S. GB	0.06%	-1.16%	1.27%	0.459	>.99	
	M.S. SF	vs.	H.S. SF	-0.18%	-1.69%	1.32%	0.569	>.99	
	H.S. GB	vs.	H.S. SF	-0.24%	-1.49%	1.01%	0.472	>.99	
V3	M.S. GB	vs.	M.S. SF	-3.38%	-8.56%	1.80%	1.958	.51	
	M.S. GB	vs.	H.S. GB	-5.68%	-9.61%	-1.74%	1.487	.001	**
	M.S. GB	vs.	H.S. SF	-7.99%	-13.29%	-2.70%	2.004	<.001	***
	M.S. SF	vs.	H.S. GB	-2.30%	-7.16%	2.57%	1.841	>.99	
	M.S. SF	vs.	H.S. SF	-4.61%	-10.64%	1.41%	2.278	.26	
	H.S. GB	vs.	H.S. SF	-2.32%	-7.31%	2.68%	1.889	>.99	
V4	M.S. GB	vs.	M.S. SF	-7.20%	-12.88%	-1.53%	2.146	.005	**
	M.S. GB	vs.	H.S. GB	-3.36%	-7.67%	0.95%	1.630	.24	
	M.S. GB	vs.	H.S. SF	-4.96%	-10.77%	0.85%	2.196	.15	
	M.S. SF	vs.	H.S. GB	3.84%	-1.49%	9.18%	2.017	.34	
	M.S. SF	vs.	H.S. SF	2.24%	-4.36%	8.85%	2.497	>.99	
	H.S. GB	vs.	H.S. SF	-1.60%	-7.07%	3.88%	2.071	>.99	

a = adjusted for multiple comparisons using Bonferroni correction.

Mean diff = mean difference based on the estimated marginal means, CI = confidence interval, LB = lower bound, UB = upper bound, SE = standard error, P-value = levels (p-lvl): * = $p < 0.05$; ** = $p < 0.01$; and *** = $p < 0.001$. No. = Number, % = Percentage. V2 = mouth is uncovered; V3 = mouth and nose are uncovered; and V4 = nose is uncovered. M.S. = middle school (students aged 12.8 ± 1.3 years old); H.S. = 4-year high school (students aged 16.7 ± 1.2 years old); GB = classes with a general school branch; and SF = school classes with a sports focus.
